# Hepatic cysteine sulphinic acid decarboxylase depletion and defective taurine metabolism in a rat partial nephrectomy model of chronic kidney disease

**DOI:** 10.1186/s12882-021-02442-7

**Published:** 2021-07-05

**Authors:** Nima Abbasian, Maryam Ghaderi-Najafabadi, Emma Watson, Jeremy Brown, Li Yu si, Debbie Bursnall, Izabella Pawluczyk, Anne-Marie Seymour, Alan Bevington

**Affiliations:** 1 Department of Respiratory Sciences, University of Leicester, Leicester, LE1 7RH UK; 2 School of Life and Medical Sciences, University of Hertfordshire, Hertfordshire, UK; 3 Department of Cardiovascular Sciences, University of Leicester, Leicester, UK; 4 Division of Biomedical Services, University of Leicester, Leicester, UK; 5 School of Life Sciences (Biomedical), University of Hull, Hull, UK

**Keywords:** Chronic kidney disease, Cysteine sulphinic acid decarboxylase, L-glutamine, Sulphinoalanine decarboxylase, Taurine

## Abstract

**Background:**

Taurine depletion occurs in patients with end-stage chronic kidney disease (CKD). In contrast, in the absence of CKD, plasma taurine is reported to increase following dietary L-glutamine supplementation. This study tested the hypothesis that taurine biosynthesis decreases in a rat CKD model, but is rectified by L-glutamine supplementation.

**Methods:**

CKD was induced by partial nephrectomy in male Sprague-Dawley rats, followed 2 weeks later by 2 weeks of 12% w/w L-glutamine supplemented diet (designated NxT) or control diet (NxC). Sham-operated control rats (S) received control diet.

**Results:**

Taurine concentration in plasma, liver and skeletal muscle was not depleted, but steady-state urinary taurine excretion (a measure of whole-body taurine biosynthesis) was strongly suppressed (28.3 ± 8.7 in NxC rats versus 78.5 ± 7.6 μmol/24 h in S, *P* < 0.05), accompanied by reduced taurine clearance (NxC 0.14 ± 0.05 versus 0.70 ± 0.11 ml/min/Kg body weight in S, P < 0.05). Hepatic expression of mRNAs encoding key enzymes of taurine biosynthesis (cysteine sulphinic acid decarboxylase (CSAD) and cysteine dioxygenase (CDO)) showed no statistically significant response to CKD (mean relative expression of CSAD and CDO in NxC versus S was 0.91 ± 0.18 and 0.87 ± 0.14 respectively). Expression of CDO protein was also unaffected. However, CSAD protein decreased strongly in NxC livers (45.0 ± 16.8% of that in S livers, *P* < 0.005). L-glutamine supplementation failed to rectify taurine biosynthesis or CSAD protein expression, but worsened CKD (proteinuria in NxT 12.5 ± 1.2 versus 6.7 ± 1.5 mg/24 h in NxC, *P* < 0.05).

**Conclusion:**

In CKD, hepatic CSAD is depleted and taurine biosynthesis impaired. This is important in view of taurine’s reported protective effect against cardio-vascular disease - the leading cause of death in human CKD.

**Supplementary Information:**

The online version contains supplementary material available at 10.1186/s12882-021-02442-7.

## Background

There is considerable evidence that both acute kidney injury and chronic kidney disease (CKD) lead in humans and in animal models to secondary defects in distant organs [1] including the liver [1–3]. The mechanism of this coupling between kidney and liver is poorly understood, but the chronic systemic inflammation that occurs in CKD may exert effects on liver [3], and the stress-activated MAP kinase P38 MAPK has been shown to mediate inflammatory effects, and also promote further release of inflammatory cytokines, in several models of inflammatory liver injury [4–9]. It has been proposed [10, 11] that the hepatic metabolic pathways that are affected during CKD include the catabolism pathways of the sulphur amino acids L-methionine and L-cysteine, which normally culminate in generation of sulphurous and sulphuric acid, and the sulphur amino acid taurine [12–14]. An abnormally low concentration of taurine has been reported in the blood plasma of end-stage CKD patients receiving dialysis therapy [10, 11]. It has been speculated that this arises from insufficiency of the enzyme cysteine sulphinic acid decarboxylase (CSAD) which plays a role in taurine biosynthesis by catalysing the conversion of cysteine sulphinic acid (CSA) to hypotaurine which is the immediate precursor of taurine [13]. Another enzyme important here is cysteine dioxygenase (CDO) upstream of CSAD which generates CSA, and is regarded as a further site of metabolic control over sulphur amino acid catabolism in liver [13]. An alternative pathway through the enzyme cysteamine (2-aminoethanethiol) dioxygenase also exists, but is regarded as a minor contributor to taurine biosynthesis [15].

Taurine depletion in CKD is undesirable [10] in view of taurine’s physiological importance [16], notably its reported protective effect [17] against endothelial dysfunction and inflammatory cardio-vascular disease which is the leading cause of death in patients with advanced CKD [18]. It has been proposed that taurine depleted patients with CKD could be treated by giving a taurine supplement, for example in dialysis fluid [19] or as an oral supplement [20]. However, such supplementation has been reported to be hazardous, as it may be difficult to control in CKD patients, leading to severe hypertaurinaemia [20]. If the impaired taurine biosynthesis suggested above is the cause of taurine depletion in CKD, an alternative approach with less risk of accidental hypertaurinaemia might be to rectify the impaired biosynthesis. The plasma concentration of taurine, both in rats and humans with intact renal function, has been reported to increase in response to dietary supplementation with L-glutamine [21] and the inward flux of taurine into liver, kidneys and gut was also reported to increase in the L-glutamine supplemented rats in that study [21]. However, whether L-glutamine supplementation can exert similar beneficial effects on taurine in the presence of CKD is unknown. L-glutamine is also a potentially attractive supplement to test in CKD in view of its reported (but disputed [22]) anabolic effects, notably in skeletal muscle.

The aim of this study was therefore to investigate defective taurine metabolism and its response to L-glutamine supplementation in a rat partial nephrectomy model of CKD. The hypothesis to be tested was that (a) depletion of the enzyme CSAD occurs in the liver of CKD rats compared with sham operated animals with intact renal function, and (b) that dietary L-glutamine supplementation rectifies the resulting defect in taurine metabolism.

## Methods

### Rat partial nephrectomy (Nx) model of CKD

All surgical and experimental procedures were performed subject to project licence reference PPL 80/2348 under the Animals (Scientific Procedures) Act (United Kingdom, 1986), and were approved by the University of Leicester Animal Welfare and Ethical Review Body.

The rat model of CKD (designated “Nx”) was established in 250 g male Sprague-Dawley rats using a one-stage surgical procedure as described previously [23]. All animals were obtained from Charles River Laboratories, Kent, UK. Briefly, rats were anaesthetised (3.5% isoflurane in oxygen delivered at 2 l per min), accompanied by sub-cutaneous administration of Rimadyl (Carpofen) 4 mg/Kg body weight) for post-operative analgesia. Anaesthesia was maintained during surgery using 2.5% isoflurane in oxygen at 1 l/min. The foot withdrawal reflex was tested to confirm anaesthesia. A 2 cm mid-line abdominal incision was made just below the sternum, and the left kidney was exposed and decapsulated, followed by clamping of the vasculature in the stem of the kidney with a 6 mm aneurysm clip. Approximately half (~ 0.4 g) of the kidney was excised (mainly from the cortex). Following removal of the clamping, bleeding was controlled by applying Surgicel® haemostat (BUNZL Healthcare, Coalville, UK) and the kidney remnant was repositioned in the animal. The right kidney was then externalised and decapsulated. Following ligation of the renal vasculature with a Mersilk® (Vicryl 0) non-absorbable suture (Johnson & Johnson, Maidenhead, Berkshire, UK) the kidney was excised, and 5 ml of 0.9% w/v sterile NaCl was applied in the peritoneal cavity to compensate for fluid loss during the surgery. An absorbable suture (Ethicon Vicryl® 3.0, Johnson & Johnson, Maidenhead, Berkshire, UK) was then used to close the abdominal muscle layer, followed by closure of the skin with an Ethicon Vicryl 5/0 suture (Johnson & Johnson, Maidenhead, Berkshire, UK).

Sham-operated control rats (designated “S”) were treated as above, with exposure and decapsulation of both kidneys, but no excision of tissue.

### Study design

Following a 14 day post-surgery recovery period on control diet containing 14.38% crude protein and no taurine (Rat and Mouse No 1 Maintenance diet expanded form RM1(E) (Product Code 801002, Special Diets Services, Essex, UK) with water ad libitum, rats were assigned for a 14 day period to three experimental groups (*n* = 8 in each group) designated NxC (partially nephrectomised CKD rats receiving control diet), NxT (partially nephrectomised CKD rats receiving test diet supplemented with L-glutamine), and S (sham-operated control animals receiving control diet).

#### Test diet

Animals in the NxT group received the control diet above supplemented with 12% L-glutamine by weight [21]. In the previously reported study [21], an isocaloric isonitrogenous supplement of control amino acids was administered to the control rats that were not receiving the L-glutamine supplement. This controlled for the effect of displacement of other nutrients in each Kg of diet by the substantial mass of L-glutamine. However, in the context of CKD, the nitrogen load associated with L-glutamine supplementation may be detrimental by worsening azotaemia. To allow the magnitude of this effect of L-glutamine to be assessed in this CKD model, the control CKD rats in Group NxC received control diet without a supplement of control amino acids.

Throughout the 14 day experimental period, the rats were housed individually in sets of 3 adjacent cages with one NxC, one NxT and one S rat in each set. This was done to minimise systematic effects of environmental differences (e.g. small gradients in temperature, humidity or lighting) across the facility in which the animals were housed. The S rat in each set was pair fed with the accompanying NxC and NxT rats. Water was provided ad libitum. On the last day of the experimental period the rats were housed in metabolic cages to allow collection of urine. At the end of the urine collection, the animals were killed by exsanguination under terminal anaesthesia. Blood was collected by aortic cannulation into heparinised containers, and organs were removed, weighed and frozen in liquid nitrogen.

### Assessment of renal and liver function

Creatinine and urea in urine and plasma were assayed colorimetrically using QuantiChrom® assay kits (Universal Biologicals, Cambridge, UK) according to the manufacturer’s instructions. Total urinary protein was determined by precipitation with trichloro-acetic acid followed by a colorimetric biuret assay [24]. Sulphate (SO_4_^2−^) excretion in urine was assayed (following removal of urinary protein by precipitation with trichloro-acetic acid), by addition of barium chloride and assay of the resulting precipitate of barium sulphate by turbidimetry [25]. The liver function test enzyme alanine transaminase (ALT) was assayed in plasma using a colorimetric catalytic assay kit (Sigma-Aldrich, Gillingham, UK) according to the manufacturer’s instructions.

Renal clearance of creatinine and taurine (expressed as ml/min/Kg body weight) was calculated using the following formula [23]:

$$ \left(\left({\mathrm{C}}_{\mathrm{u}}/{\mathrm{C}}_{\mathrm{p}}\right)\ \mathrm{x}\ {\mathrm{V}}_{\mathrm{u}}\right)/\mathrm{BW} $$

where C_u_ is the concentration of the solute in urine, C_p_ is the concentration of the solute in plasma, V_u_ is the average rate of urine production (in ml per minute) during the urine collection period, and BW is the body weight in Kg.

### Assessment of body composition

Twenty mg samples of frozen liver were homogenised in 800 μl of ice-cold 0.3 M perchloric acid and centrifuged for 10 min at 4 °C and 3000 x *g* to sediment acid-insoluble material which was assayed for total protein [26] and total DNA [27]. Acid in the supernatant was neutralised by vortexing with an equal volume of tri-n-octylamine: 1,1,2-trichloro, trifluoro-ethane (22:78 vol/vol) [28] and the top (aqueous) phase was removed and stored at − 80 °C for subsequent analysis of free amino acids. Free amino acids (including taurine) were subjected to pre-column derivatization with o-phthalaldehyde/3-mercaptopropionate/9-fluorenylmethylchloroformate, and the derivatives were separated on an Agilent 1100 high-performance liquid chromatograph with a Zorbax Eclipse AAA (4.6 × 75 mm, 3.5 μm) column at 40 °C and quantified by post-column ultraviolet detection. As skeletal muscle contains the body’s largest pool of lean tissue and of free amino acids (including taurine), the same procedures were also applied to fast-twitch muscle (gastrocnemius) and slow-twitch muscle (soleus) from each rat.

Amino acids in plasma were assayed following deproteinisation of 500 μl aliquots of plasma with 12.5 μl aliquots of 12 M perchloric acid and neutralisation as described above.

### RNA extraction and qRT-PCR

Total RNA was extracted using TRIzol reagent (Invitrogen, Paisley, UK). Using 1 μg total RNA, cDNA was synthesized using an AMV reverse transcription system (Promega, Southampton, UK) according to the manufacturer’s instructions. Forward and reverse primer sequences used in this study are listed in Table 1. Real-time PCR was performed using an Applied Biosystems 7500 Fast Real-Time PCR System (Thermo Fisher Scientific, Loughborough, UK) using SYBR green PCR reagent (Thermo Fisher Scientific, Loughborough, UK). Relative amounts of mRNA were normalized to the corresponding cyclophilin A signal for each sample, and relative expression is presented as (2^-ΔΔCT^) [29].

**Table 1:** Primers used for qRT-PCR

Gene	Primer sequence	
CSAD	Forward 5′ – 3′	AGATGATCCCTGAGGATCTG
	Reverse 5′ – 3′	TGGATCCCATCCAGGAGATG
CDO	Forward 5′ – 3′	GACTGGCCTGACAAGAAATC
	Reverse 5′ – 3’	TACAAGTGAAGGCTCACAGC
TauT (SLC6A6)	Forward 5′ – 3’	TCTTCATTGCCATCGTGTGC
	Reverse 5′ – 3’	AGGCATACACCACTAGCTGC
GAT2 (SLC6A13)	Forward 5′ – 3’	GAAGAACCGGAGGGAGATTC
	Reverse 5′ – 3’	GAGAACAGGAAGGTTGCCAG
Cyclophilin A	Forward 5′ – 3’	CACCGTGTTCTTCGACATC
	Reverse 5′ – 3’	TGCTGTCTTTGGAACTTTGTC

### Immunoblotting

Cell lysates from each rat were subjected to SDS-PAGE (30 μg protein per lane) followed by immunoblotting. Immunoblotting was performed onto nitrocellulose membranes (GE-Healthcare, Little Chalfont, UK) followed by probing with primary antibodies against: dually phosphorylated (P-Thr^183^/P-Tyr^185^) JNK2 (Promega, Southampton, UK); P38 MAPK (Cell Signaling Technology, London, UK); phosphorylated (P-Thr^180^/P-Tyr^182^) P38 MAPK (Cell Signaling Technology, London, UK); β-Actin (Sigma-Aldrich, Gillingham, UK); CSAD (custom antibody raised against peptide [C]-SLRGKKESPDYSQRLS-amide) (Cambridge Research Biochemicals, Billingham, UK); and CDO (a kind gift from Prof Martha Stipanuk, Cornell University, New York, USA). Polyclonal Rabbit Anti-Mouse and Goat Anti-Rabbit Immunoglobulins/HRP (DakoCytomation (Agilent), Santa Clara, USA) were used as secondary antibodies as appropriate and HRP-labelled proteins were detected by chemiluminescence using ECL reagent (GE-Healthcare, Little Chalfont, UK). Band intensities were quantified by Image Lab Software v 5.2.1 (Bio-Rad) and data are presented as the ratio of the intensity for the protein of interest/housekeeping protein expressed as a % of the corresponding ratio under control conditions.

### Statistical analysis

Analysis was performed using GraphPad Prism 8.1.2. Normally distributed data are expressed as Mean ± SEM and were analysed by 1-way repeated measures ANOVA on the matched-fed groups, with Tukey’s range test post hoc testing. Non-normally distributed data (determined by the Shapiro-Wilk test) are expressed as Median (Inter-quartile Range) and were analysed by Friedman’s 1-way ANOVA with Dunn’s multiple comparisons post hoc testing.

## Results

### Confirmation of the CKD model

The animals in the 3 experimental groups were matched for initial body weight and weight of food consumed over the 14 day experimental period (Table 2). Comparison of the partially nephrectomised rats in Groups NxC and NxT with the sham-operated rats in Group S showed moderately reduced glomerular filtration rate (GFR) (assessed from creatinine clearance) and moderate azotaemia (elevated plasma concentrations of urea and creatinine) (Table 2). Proteinuria also occurred, but only in the L-glutamine supplemented NxT animals (Table 2). In this short 14 day study, no significant cachexia was observed, judged from Day 14 body weight, body weight gain over the 14 days, skeletal muscle weights, heart weight or liver weight (Table 2). Assessment of protein mass in relation to cell number in these tissues, using the total protein/DNA ratio, also failed to detect significant cachexia (Table 2).

**Table 2:** Characterisation of the CKD model

	Sham-operated, on Control diet, S (*n* = 8)	Partial nephrectomy, on Control diet, NxC (*n* = 8)	Partial nephrectomy, on Test diet, NxT (*n* = 8)
Body composition			
Body weight (Day minus 14) (g)	313.0 ± 14.5	308.9 ± 14.8	307.1 ± 15.1
Body weight (Day 1) (g)	335.6 ± 15.9	305.3 ± 13.2*	305.3 ± 13.7
Body weight (Day 14) (g)	374.0 ± 13.4	345.8 ± 12.2*	333.0 ± 12.8*
Body weight gain over days 1–14 (g)	38.4 ± 5.6	40.5 ± 5.0	27.7 ± 4.2^|^
Food consumed over days 1–14 (g)	317.5 ± 12.3	320.0 ± 12.6	312.7 ± 11.1
Gastrocnemius (mean of left & right)			
Wet weight (g)	2.43 ± 0.08	2.25 ± 0.07	2.16 ± 0.12
Protein (μg per μg of DNA)	142 (121–182)	140 (111–164)	184 (117–281)
Soleus (mean of left & right)			
Wet weight (g)	0.171 ± 0.013	0.163 ± 0.006	0.180 ± 0.023
Protein (μg per μg of DNA)	62 (50–71)	58 (50–76)	59 (43–65)
Heart			
Wet weight (g)	1.237 ± 0.054	1.109 ± 0.042	1.192 ± 0.045
Protein (μg per μg of DNA)	139 (67–223)	120 (46–197)	137 (72–457)
Liver			
Wet weight (g)	12.27 ± 0.88	10.55 ± 0.29	11.28 ± 0.63
Protein (μg per μg of DNA)	35 (32–42)	35 (26–85)	30 (25–61)
Left kidney			
Wet weight (g)	1.217 ± 0.041	1.268 ± 0.064	1.374 ± 0.099
Plasma parameters			
Creatinine (μM)	38.44 ± 2.56	92.60 ± 7.95*	115.65 ± 29.06
Urea (mM)	4.36 ± 0.72	13.27 ± 1.05*	26.44 ± 4.23*¶
24 h Urinary excretion parameters			
Creatinine (μmol/24 h)	77.18 ± 8.04	68.89 ± 9.17	63.67 ± 7.39
Urea (mmol/24 h)	9.72 ± 0.94	10.45 ± 1.56	25.11 ± 2.76*^|^
Total protein (mg/24 h)	6.46 ± 0.71	6.74 ± 1.50	12.49 ± 1.19*^|^
Total volume (ml/24 h)	14.64 ± 1.31	23.71 ± 1.94*	53.45 ± 4.57*^|^
Creatinine clearance (GFR) (ml/min/Kg body weight)	4.06 ± 0.47	1.51 ± 0.17*	1.51 ± 0.35

Unless otherwise stated, all parameters refer to Day 14 (i.e. 28 days after surgery). Feeding of experimental diets was performed from Day 1 to Day 14. Data are presented as Mean ± SEM or as Median (inter-quartile range). *P < 0.05 versus Sham; ^|^*P* < 0.05 versus NxC ¶*P* = 0.064 versus NxC

### Confirmation of L-glutamine supplementation

Even though no significant increase in the concentration of free L-glutamine or its catabolite L-glutamate was detected in the plasma or tissues of the L-glutamine-treated NxT rats compared with the NxC group (Table 3), significantly enhanced urea excretion was observed in the urine of the NxT rats (Table 2) and measurement of urine volume showed significant diuresis in the NxT group (Table 2). No significant anabolic effect of L-glutamine was observed, judged from Day 14 body weight, 14 day body weight gain, skeletal muscle weights, heart weight, liver weight or total protein/DNA ratio in these tissues (Table 2). The nitrogen load accompanying this L-glutamine supplement also resulted in apparent worsening of azotaemia (plasma urea in Table 2) although this fell short of statistical significance (*P* = 0.064 versus NxC). No statistically significant difference in residual kidney mass was observed between the NxT group and the NxC group (Table 2).

**Table 3:** Effect of CKD and dietary L-glutamine supplementation on free amino acids and sulphate (SO_4_^2−^)

	Sham-operated, on Control diet, S (*n* = 8)	Partial nephrectomy, on Control diet, NxC (*n* = 8)	Partial nephrectomy, on Test diet, NxT (*n* = 8)
Tissue parameters			
Gastrocnemius (mean of left & right)			
L-glutamine (μmol/g)	5.48 ± 1.00	6.23 ± 1.31	5.61 ± 0.87
L-glutamate (μmol/g)	1.20 ± 0.44	1.38 ± 0.36	1.51 ± 0.47
L-cysteine (nmol/g)	44 ± 14	31 ± 13	27 ± 15
L-methionine (nmol/g)	82 ± 34	98 ± 36	100 ± 27
Taurine (μmol/g)	16.4 ± 2.7	15.6 ± 2.1	14.8 ± 1.2
Soleus (mean of left and right)			
L-glutamine (μmol/g)	12.0 ± 2.7	15.1 ± 4.5	18.1 ± 6.1
L-glutamate (μmol/g)	3.89 ± 1.25	5.51 ± 1.53	6.78 ± 1.75
L-cysteine (nmol/g)	ND	ND	ND
L-methionine (nmol/g)	99 ± 30	96 ± 23	156 ± 53
Taurine (μmol/g)	25.9 ± 2.6	25.7 ± 1.7	25.2 ± 1.8
Liver			
L-glutamine (μmol/g)	7.11 ± 1.04	8.10 ± 1.13	9.09 ± 1.98
L-glutamate (μmol/g)	2.70 ± 0.89	1.76 ± 0.54	2.29 ± 0.50
L-cysteine (nmol/g)	ND	ND	ND
L-methionine (nmol/g)	139 ± 20	101 ± 16	208 ± 45
Taurine (μmol/g)	5.04 ± 1.51	8.21 ± 1.84	8.05 ± 1.71
Plasma parameters			
L-glutamine (μM)	830 ± 63	1067 ± 163	902 ± 228
L-glutamate (μM)	92.6 ± 23.8	156.3 ± 36.9	174.0 ± 46.4
L-cysteine (μM)	29.9 ± 4.2	46.3 ± 11.6	41.0 ± 8.4
L-methionine (μM)	70.9 ± 18.1	94.1 ± 19.1	79.9 ± 15.8
Taurine (μM)	289 ± 93	486 ± 119	354 ± 135
24 h Urinary excretion parameters			
L-glutamine (μmol/24 h)	0.42 ± 0.22	4.81 ± 2.43	17.88 ± 5.62*
L-glutamate (μmol/24 h)	2.52 ± 0.33	1.72 ± 0.56	4.63 ± 0.99^|^
Taurine (μmol/24 h)	78.48 ± 7.63	28.25 ± 8.65*	15.56 ± 3.67*
Sulphate (SO_4_^2−^) (μmol/24 h)	159.0 ± 9.8	155.2 ± 18.7	147.8 ± 30.5
Taurine/sulphate (SO_4_^2−^) molar ratio	0.507 ± 0.054	0.181 ± 0.048*	0.138 ± 0.041*
Total taurine + sulphate (SO_4_^2−^) (μmol/24 h)	237.5 ± 14.0	183.4 ± 22.1*	163.4 ± 33.1*
Taurine clearance (ml/min/Kg body weight)	0.703 ± 0.105	0.136 ± 0.053*	0.133 ± 0.068*

All parameters refer to Day 14 (i.e. 28 days after surgery). Feeding of experimental diets was performed from Day 1 to Day 14. Concentrations in tissue are expressed as nmol or μmol/g wet weight. Data are presented as Mean ± SEM. *P < 0.05 versus Sham. ^|^*P* < 0.05 versus NxC

*ND* Not detectable

A small increase in the urinary excretion of free L-glutamine and L-glutamate was detected in the NxT rats compared with the NxC group (Table 3) but, when expressed as moles of nitrogen per 24 h, this increase was only about 0.1% of the corresponding increase in urinary urea nitrogen (Table 2).

### Effect of the CKD model on taurine and sulphate

Even though the diet fed to all three experimental groups in this study contained no taurine, a significant rate of urinary taurine excretion was detected in all of the rats (Table 3). Comparison of this rate in the NxC CKD group with the control rate in sham operated group S, revealed a significant 64% fall in this taurine excretion rate in CKD and the urinary taurine / SO_4_^2−^ ratio was also significantly decreased by 64% in the NxC group compared with the S group (Table 3). The total rate of sulphur excretion (taurine plus SO_4_^2−^) was 23% lower in the NxC group compared with the S group (Table 3). Mean total taurine plus SO_4_^2−^ excretion rate was also 11% lower in the NxT group than in the NxC group (Table 3).

In spite of the significant decline in the steady-state rate of appearance of taurine in the urine in the CKD rats (suggesting impaired taurine biosynthesis - see Discussion), there was no corresponding fall in the concentration of taurine in plasma, in liver or in muscle (Table 3). When the plasma taurine concentration and the rate of urinary taurine excretion were used to calculate taurine clearance (Table 3), this was found to be 81% lower in the NxC and NxT groups of CKD animals compared with the sham-operated control rats.

Measurements of L-methionine (Table 3) failed to detect any reproducible changes in plasma, liver or muscle in the NxC group compared with the S group that might have contributed to downstream effects on taurine. Similarly no significant changes in L-cysteine were observed in plasma and in gastrocnemius.

### Effect of CKD on liver

**Fig. 1:**
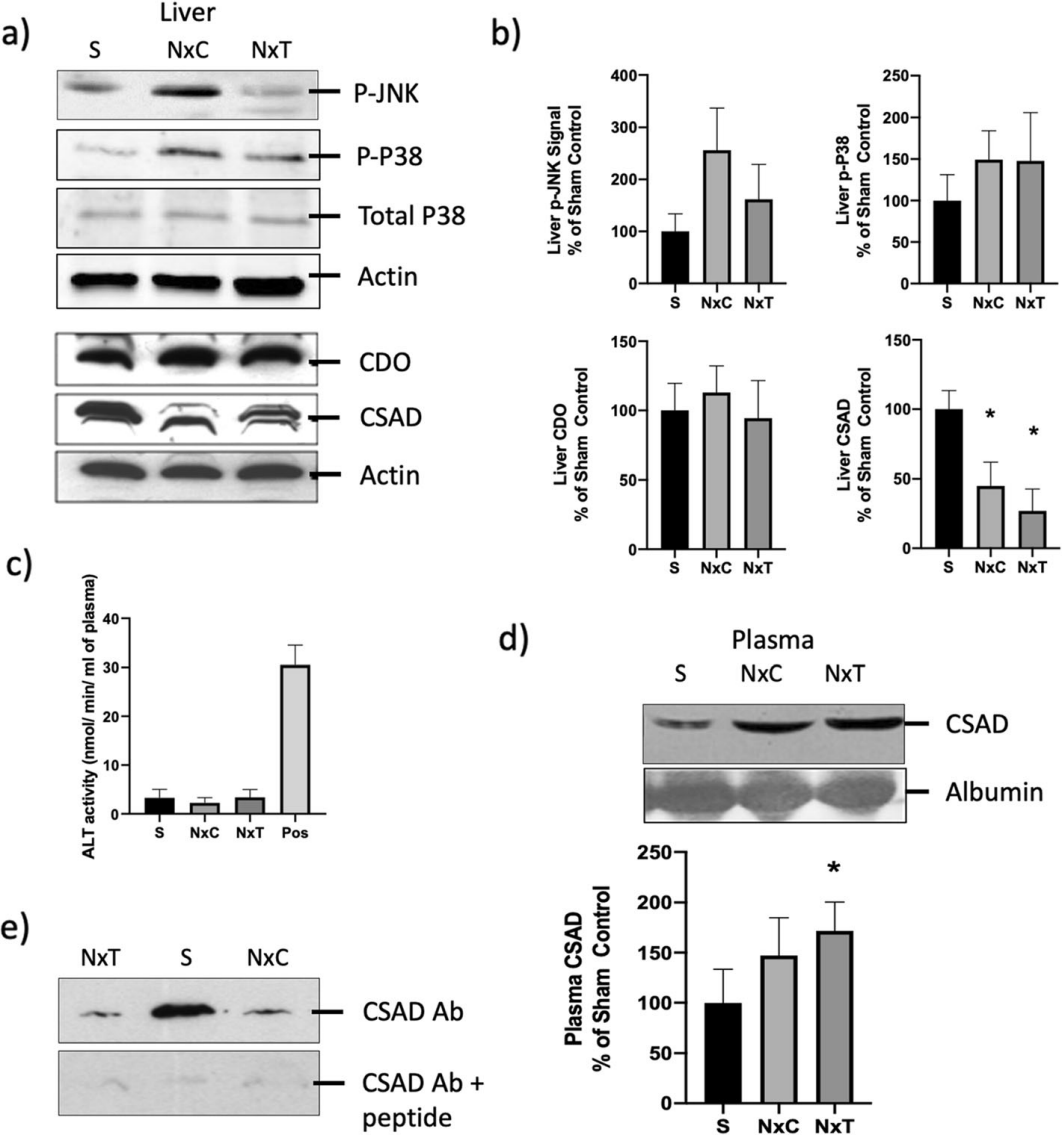
Response of rat liver to CKD with (NxT) or without (NxC) dietary L-glutamine supplementation. S denotes sham-operated control rats. All data refer to Day 14 (i.e. 28 days after surgery). Feeding of experimental diets was performed from Day 1 to Day 14. **a** Representative immunoblots obtained from liver lysates using antibodies against P-JNK (dually phosphorylated (P-Thr^183^/P-Tyr^185^) JNK2); P-P38 (phosphorylated (P-Thr^180^/P-Tyr^182^) P38 MAPK); Total P38 MAPK; β-Actin; CDO (cysteine dioxygenase); and CSAD (cysteine sulphinic acid decarboxylase). **b** Quantification of blots from (**a**) by densitometry. **c** Colorimetric catalytic assay for alanine transaminase (ALT) in plasma. POS denotes reading obtained with positive control provided in the assay kit. **d** Representative immunoblot obtained from blood plasma using antibody against CSAD, with albumin as loading control (top panel) and quantification of blots by densitometry (bottom panel). **e** CSAD immunoblot showing the effect of incubation with primary anti-CSAD antibody as in (**a**) (top panel); or overnight pre-incubation of primary antibody with antigenic blocking peptide [C]-SLRGKKESPDYSQRLS-amide at 10 μg/ml (bottom panel). Data are presented as Mean ± SEM, *n* = 8 rats in each group. *P < 0.05 versus Sham

When liver lysates were probed by immunoblotting, a tendency towards phospho-activation of the stress-activated MAP kinases JNK and P38 was detected in the CKD rats in the NxC group relative to the sham-operated control rats (Fig. 1a, b). However this did not reach statistical significance (*P* = 0.072 for P-JNK; *P* = 0.278 for P-P38). Furthermore, assay of the liver function test (LFT) enzyme alanine transaminase (ALT) in the rats’ plasma detected no increase in the CKD animals (Fig. 1c).

qRT-PCR detected strong expression in liver of mRNAs encoding both CDO and CSAD, the two principal enzymes that regulate sulphur amino acid catabolism (Table 4). CKD had no statistically significant impact on the expression of CDO or CSAD mRNA (Table 4), but did up-regulate expression of the mRNA encoding taurine transporters GAT2 and TauT (Table 4).

**Table 4:** Effect of CKD and dietary L-glutamine supplementation on expression of mRNAs encoding regulatory enzymes of taurine biosynthesis and taurine transporters in rat liver on Day 14 (i.e. 28 days after surgery)

	Sham-operated, on Control diet, S (*n* = 8)	Partial nephrectomy, on Control diet, NxC (*n* = 8)	Partial nephrectomy, on Test diet, NxT (*n* = 8)
Liver			
CSAD	1.19 ± 0.35	0.91 ± 0.18	0.33 ± 0.13
CDO	1.08 ± 0.20	0.87 ± 0.14	1.00 ± 0.14
TauT (SLC6A6)	0.81 (0.64–0.90)	3.38 (1.86–4.33)	3.50 (1.71–15.14)*
GAT2 (SLC6A13)	0.95 (0.87–1.08)	2.16 (1.22–14.64)	22.7 (19.73–32.84)*

Feeding of experimental diets was performed from Day 1 to Day 14. Relative amounts of mRNA were normalized to the corresponding cyclophilin A signal for each sample, and relative expression is presented as (2^-ΔΔCT^). Data are presented as Mean ± SEM or as Median (inter-quartile range). *P < 0.05 versus Sham

In contrast, when expression of CSAD protein was assessed by immunoblotting in the livers of the NxC group of CKD rats, this showed a clear decrease to 45% of the value in sham operated control animals (S) (Fig. 1a, b), consistent with the reduction in the taurine flux into urine in the NxC group (36% of the S value in Table 3) and the accompanying reduction in the urinary taurine/ SO_4_^2−^ excretion ratio (36% of the S value in Table 3). Conversely, immunoblotting performed on plasma detected an apparent increase in the CSAD protein signal (Fig. 1d). Specificity of the signal observed in the CSAD immunoblots was confirmed by abolition of the signal (both in liver (Fig. 1e) and in plasma) on pre-incubating the primary antibody with the specific CSAD blocking peptide. Probing for expression of CDO protein in the same livers detected no change in response to CKD (Fig. 1a).

### Impact of L-glutamine on taurine in this CKD model

In spite of the previously reported increase in plasma taurine concentration induced by L-glutamine in rats with intact renal function [21], dietary supplementation with L-glutamine failed to increase the taurine concentration in plasma in the present CKD model (Table 3), nor did it normalise the taurine / SO_4_^2−^ ratio in urine (Table 3), or the CSAD protein expression in liver (Fig. 1a, b). Indeed the mean level of expression of CSAD mRNA seemed lower in the L-glutamine supplemented NxT group than in S and NxC (relative expression 0.33 ± 0.13 for NxT in Table 4), but this was not statistically significant.

## Discussion

### Characterisation of the CKD model and L-glutamine supplementation

In good agreement with earlier characterisation of this surgical model of CKD [23], the partially nephrectomised rats in groups NxC and NxT in the present study showed reduced GFR and moderate azotemia when compared with the sham-operated control animals (Table 2). In spite of failure to detect an increase in the plasma or tissue L-glutamine concentration in the L-glutamine supplemented NxT group (Table 3), there was good evidence for catabolism of the ingested L-glutamine resulting in enhanced urea excretion and consequently urea-induced osmotic diuresis (Table 2) which has previously been reported as a consequence of enhanced urea production in rats [30]. In spite of this, no significant anabolic effect of L-glutamine was observed (Table 2), indeed the 14 day body weight gain was lower in the NxT rats compared with the NxC group, possibly as a consequence of diuresis. Furthermore, in spite of the increased ureagenesis in the NxT rats, no significant increase in residual renal mass was observed in comparison with the NxC group (Table 2) in contrast with the urea-induced renal hypertrophy that has previously been reported in urea-supplemented rats with intact kidneys [30].

### Evidence of defective taurine metabolism

The main strength of the present study is that it is the first to demonstrate by direct measurements in a rat model of CKD the hepatic depletion of CSAD, a key enzyme of taurine biosynthesis, accompanied by a marked reduction in the steady-state rate of appearance of taurine in the animals’ urine and a decrease in the taurine/sulphate excretion ratio (Fig. 1 and Table 3).

It has been suspected for more than 20 years that a defect exists in the hepatic biosynthesis of taurine in human patients with advanced CKD, both in pre-dialysis patients [11] and in end-stage patients receiving dialysis [10, 11]. The steady-state rate of appearance of taurine in the urine in the absence of dietary taurine has previously been used as a measure of the endogenous rate of taurine biosynthesis [31]. The decline in taurine excretion rate in Table 3 in the present study is therefore consistent with the impaired taurine biosynthesis arising from CSAD insufficiency that was previously proposed [10]. In liver, CSAD is the enzyme that partitions the sulphur amino acid flux between the pathway to taurine and the alternative pathway to sulphurous and sulphuric acid (which can be assessed by measuring sulphate SO_4_^2−^ excretion [31]). If the previously proposed insufficiency of CSAD in CKD [10] is occurring here, the ratio of taurine generation to SO_4_^2−^ generation would be expected to decrease. As predicted, this ratio did indeed decrease in the NxC group compared with the S group (Table 3), consistent with CSAD impairment (probably in the liver [32]).

If the metabolic flux through CSAD is impaired in the NxC group, a possible outcome is diversion of the flux of sulphur down the alternative pathway to SO_4_^2−^ resulting in increased 24 h excretion of SO_4_^2−^ in the NxC group compared with the sham-operated S group. This increase was not observed (Table 3) and, as a consequence, the total rate of sulphur excretion (taurine plus SO_4_^2−^) was lower in the NxC group compared with the S group (Table 3). The further observation that total taurine plus SO_4_^2−^ excretion rate was 11% lower in the NxT group than in the NxC group (Table 3) probably reflects the displacement of dietary sulphur amino acids in each Kg of diet by the 12% L-glutamine supplement.

### Mechanism of defective taurine metabolism in CKD

In spite of the findings above, it is important to emphasise that a defect in hepatic biosynthesis is unlikely to be the only abnormality in taurine physiology in CKD, either in the current rat model or in end-stage human CKD. The observation here in the NxC rats of unaltered taurine concentration in plasma, liver and muscle (Table 3) in spite of a significant steady-state decline in the rate of appearance of taurine in the urine demonstrated a marked renal retention of taurine, with an 81% decrease in taurine clearance. Much of this retention may be attributable to the reduced GFR in this CKD model (demonstrated by the accompanying 63% decrease in creatinine clearance (Table 2)). Such taurine retention may explain why, in non-taurine supplemented patients with end-stage renal failure, whose plasma taurine concentration is clearly low [10, 11], subsequent moderate oral taurine supplementation results in severe hypertaurinaemia that would not be observed with this level of supplementation in healthy individuals [20].

A further taurine abnormality observed here was that in the NxT group there was significantly enhanced hepatic expression of genes encoding taurine transporters TauT and GAT2 (Table 4) which perform inwardly directed active transport of taurine in hepatocytes [33, 34]. Compensatory up-regulation of the transporters to restore intra-hepatocyte taurine concentration in the face of CSAD insufficiency is a possible explanation. However, it should be noted that this gene expression effect did not reach statistical significance in the clearly CSAD-deficient NxC group (Table 4); and up-regulation of TauT mRNA was not observed in CSAD knock-out mice with normal renal function [15]. Uraemia may be a contributor to this CKD-induced transporter upregulation. This is supported by the fact that ureagenesis (shown by the high 24 h urea excretion rate) and plasma urea concentration in the NxT group were both twice as high as in the NxC group (Table 2), and may explain why the transporter up-regulation reached statistical significance in the NxT group but not in the NxC group (Table 4).

The observation here of a significant reduction in the 24 h flux of taurine into urine, implying impairment of taurine biosynthesis, could in principle have arisen from a decrease in the activity of additional enzymes other than just CSAD; for example CDO, the other major enzyme (upstream of CSAD) that influences sulphur amino acid catabolism in liver and hence the total rate of production of taurine plus sulphate (SO_4_^2−^) [13]. Even though no significant decline in CDO expression at mRNA level or at protein level was detected in the NxC rats compared with the sham-operated control rats (Table 4 and Fig. 1a), a decline in the metabolic flux through CDO (possibly because of events upstream of CDO) cannot be ruled out. The occurrence of such additional effects of CKD (independent of CSAD) may explain why the total 24 h rate of taurine + sulphate (SO_4_^2−^) excretion into urine was 23% lower in the NxC CKD group than in the sham-operated S group (Table 3). Theoretically depletion of sulphur amino acid substrates L-methionine and L-cysteine could have contributed to this decrease, but no evidence for such depletion in NxC versus S group rats was found in the plasma or tissue amino acid pools that were detectable in Table 3. Indeed, as in human skeletal muscle in an earlier report [35], free L-cysteine was not routinely detectable in soleus and liver in the present study.

There was no evidence in this study of significant transcriptional effects of CKD on CSAD expression in the NxC rats (Table 4). It therefore seems unlikely that the previously documented transcriptional regulation of CSAD by bile acids [36] was significantly disturbed here by the bile acid abnormalities that occur in end-stage CKD [37]. However, it should not be concluded from the present data that effects at mRNA level play no role in the taurine metabolism abnormalities described here. For example, even though it was not statistically significant, the marked downward trend in CSAD mRNA in the NxT group merits further investigation and it would be of interest in future work to examine the time course of CSAD and CDO mRNA in CKD.

It remains to be determined whether CKD affects post-transcriptional regulation of hepatic CSAD expression and whether this decreases the stability of the protein to proteolysis, or alters trafficking of the protein within the cell (possibly to the extracellular compartment – as implied by Fig. 1d). It has been reported that, at least in brain, CSAD is phosphorylated, a post-translational modification which activates the enzyme [38]; and more recent studies have detected phosphorylation specifically at threonine residue 84 in human CSAD [39]).

### Impact of L-glutamine supplementation

Even though the 14-day dietary L-glutamine supplement was well tolerated by the partially nephrectomised rats in this study, it yielded no obvious benefits, either in terms of rectifying the taurine defect or in terms of body composition. It also worsened proteinuria and caused a significant increase in urinary urea excretion. The resulting urea-induced osmotic diuresis is clearly undesirable, not least because this effect of urea has been reported to cause clinically significant hypernatraemia in humans [40].

### Limitations of this study

An earlier study of the effect of L-glutamine supplementation in rats with intact renal function [21] reported an increase in plasma L-glutamine and taurine concentration. Neither of these were observed in the present study. This difference may have arisen because the present study differs in two important respects from the earlier one: firstly because of the presence of CKD; secondly because the control (non-L-glutamine supplemented) rats here were not given the iso-caloric iso-nitrogenous load of control amino acids (comprising L-asparagine, L-serine, glycine, L-proline, and L-alanine) that was applied in the original study [21]. This change was made here to allow the effect of L-glutamine’s accompanying calorie and nitrogen load to be assessed in the CKD rats. Displacement of other nutrients in each Kg of diet by the substantial mass of L-glutamine could in principle have affected the taurine status of the CKD rats. However, applying a heavy control load of non-L-glutamine neutral amino acids (as in the earlier study [21]) also risked confounding effects (e.g. by competitive inhibition or down-regulation of L-methionine transporters such as SNAT2/SLC38A2 which are regulated by neutral amino acid load [41]). As sham control groups receiving the test diet or the previously described non-L-glutamine neutral amino acid load [21] were not included in the present study, it was not possible to assess the effect of L-glutamine supplementation or the non-specific effects of other neutral amino acids on taurine metabolism in the absence of CKD. In future work such controls might be helpful to shed light on why L-glutamine failed here to show the previously reported [21] beneficial effects on taurine physiology.

A further limitation in this study was that no baseline collection of urine data was obtained from the rats at the start of the 14-day experimental period or before surgery. In principle any initial mismatching in baseline amino acid and sulphur metabolism between the groups of animals might have contributed to the failure to observe here the previously reported effect of L-glutamine supplementation on taurine metabolism. It is possible therefore that some beneficial effects of L-glutamine supplementation were overlooked in the present study as a consequence of this.

Finally, even though a number of other metabolic defects have been reported in liver in CKD [1–3], possibly occurring through the P38 MAP kinase-mediated inflammatory injury that is observed in models of liver injury [4–9], measurements of P38 and JNK MAP kinase activation in the present study detected no statistically significant activation of these kinases in the NxC rats (Fig. 1a, b), nor did measurements of the LFT enzyme ALT detect any abnormality (Fig. 1c). The mechanism linking CKD to the taurine defects described in this study therefore remains to be determined.

### Implications of defective CSAD in CKD

This study is the first to report a substantial defect in the enzyme CSAD (and hence in taurine metabolism) in a pathological state (CKD) relevant to human disease. As it has been reported that taurine may play a significant role in prevention of endothelial and cardiovascular dysfunction [17], at least partly through its potent physiological [42] and anti-oxidant [16] effects, it is likely that CSAD insufficiency in CKD has pathological consequences, possibly contributing to the burden of cardiovascular disease which is the major cause of death [18] in CKD patients. This view is reinforced by evidence from CSAD knock-out mice in which there was considerable mortality in the mice accompanied by up-regulation of genes encoding anti-oxidant enzymes [15], indicating oxidative stress. Significant oxidative stress is known to occur in the present partial nephrectomy CKD model when it is extended to 12 weeks post-surgery [23]. In the present relatively short 14 day study, commencing 14 days post-surgery, renal retention of taurine maintained the taurine pool size in the CKD model (Table 3) in spite of the CSAD depletion, but long-term taurine depletion is known to occur in advanced human CKD [10, 11] implying that the taurine retention effect observed here has limited duration. For this reason, further work on the molecular basis of hepatic CSAD depletion and long-term taurine abnormalities may be of value in reducing the burden of oxidative stress and cardiovascular mortality which are of such importance in patients with CKD.

## Supplementary Information

**Additional file 1.**

## Data Availability

The datasets used and/or analysed during the current study are available from the corresponding authors on reasonable request.
