# Supplementary material for: Hepatic cysteine sulphinic acid decarboxylase depletion and defective taurine metabolism in a rat partial nephrectomy model of chronic kidney disease
Source: BMC Nephrol. 2021 Jul 5;22:250. doi: 10.1186/s12882-021-02442-7 (PMC8256558; doi:10.1186/s12882-021-02442-7)
Supplement: Supplementary file 1 — Additional file 1. [file 12882_2021_2442_MOESM1_ESM.pdf]

Fig 1a CDO

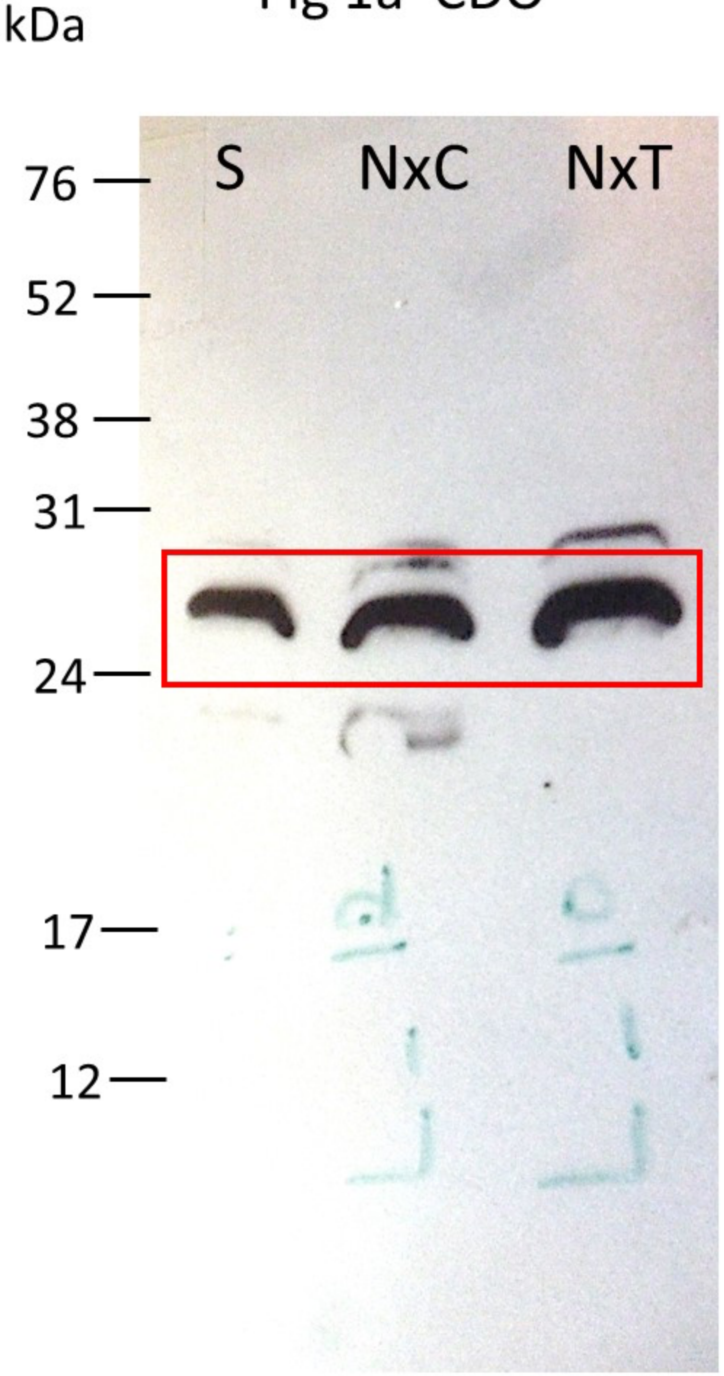

Fig 1a CSAD

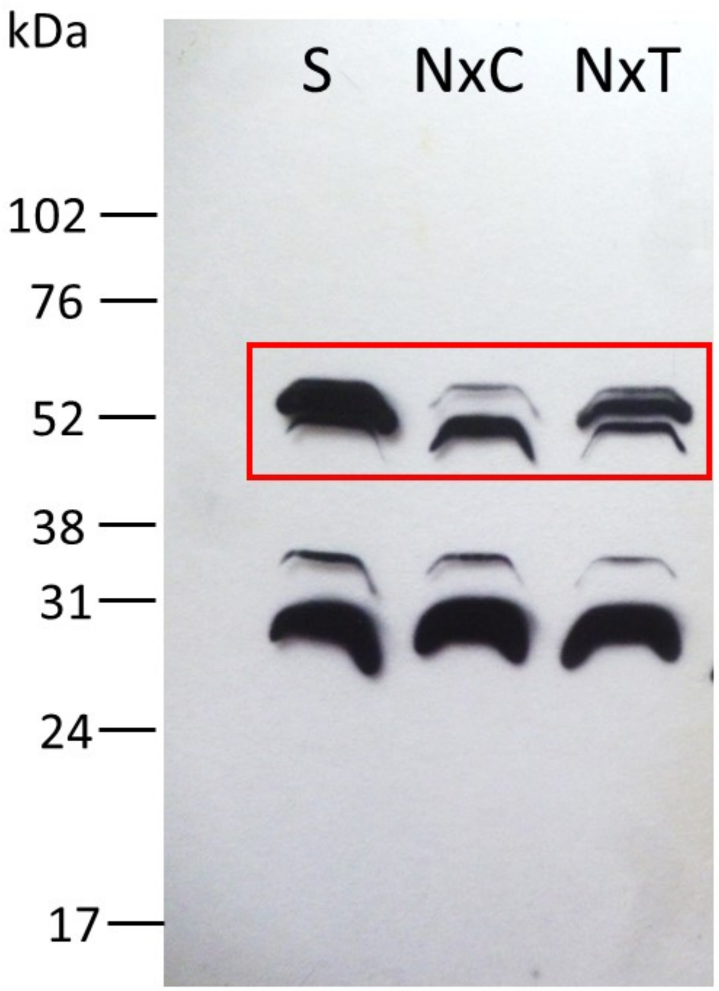

in liver lysates, the anti-CSAD antibody also detects a truncated ~ 30kDa CSAD fragment. This staining is specific (i.e. it is absent in the presence of blocking peptide) and it occurs in liver but not in plasma, possibly indicating that it is a degradation fragment generated within the hepatocytes.

Fig 1a Lower Actin Blot

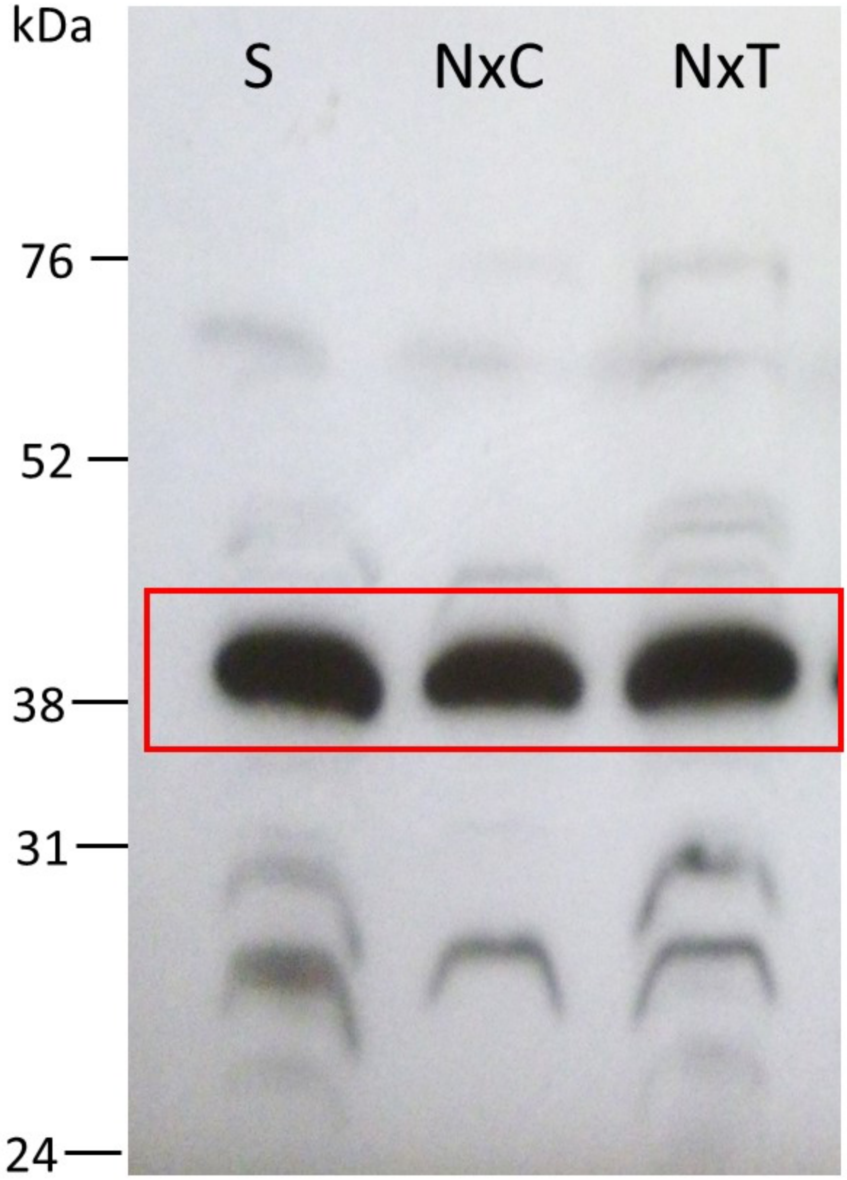

Fig 1a P-JNK

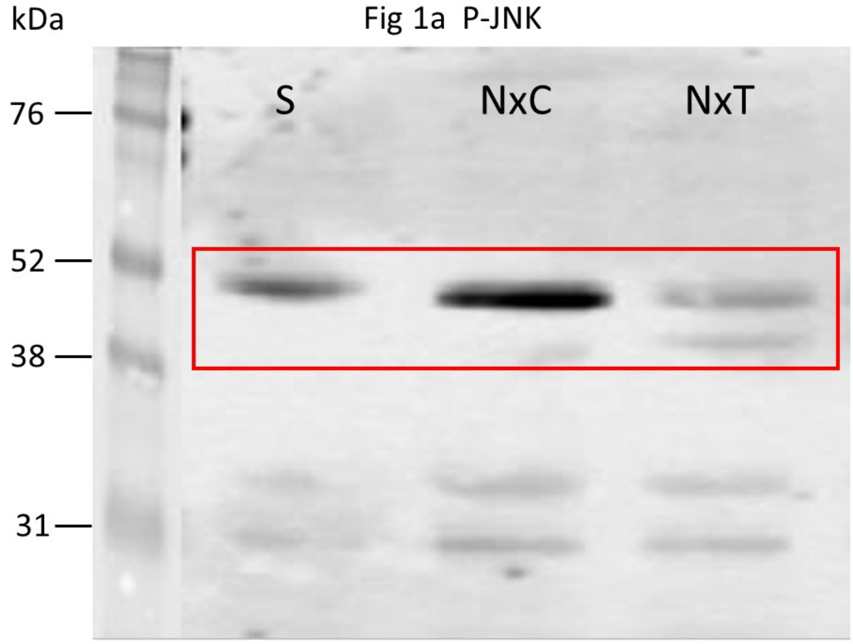

Fig 1a P-P38 in Liver

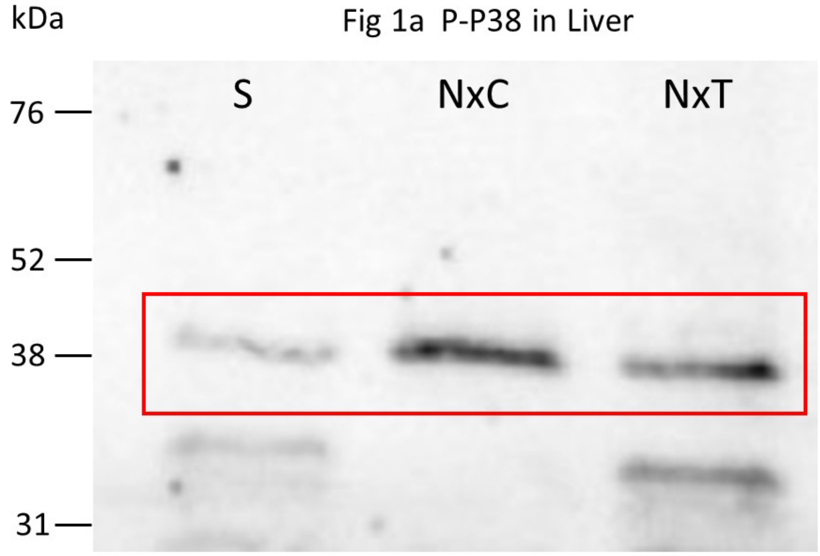

Fig 1a Total P38 in Liver

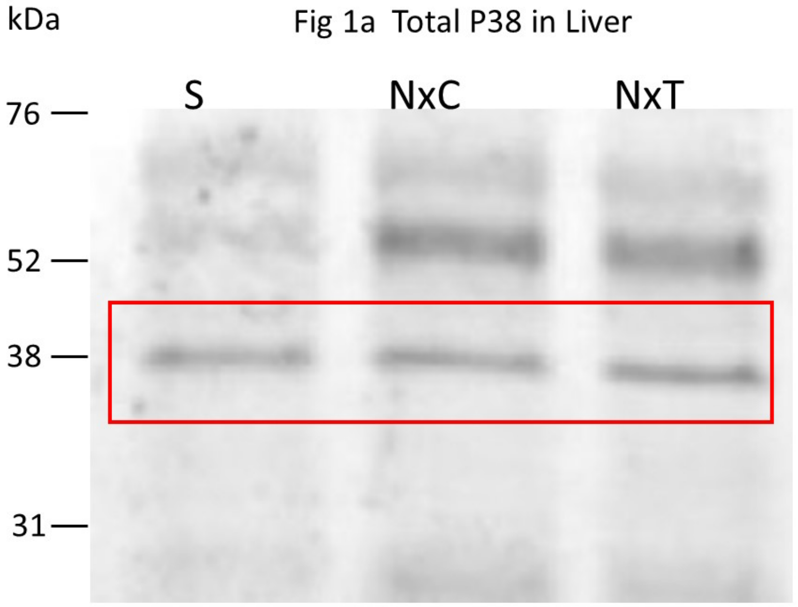

Fig 1a Upper Actin Blot in in Liver

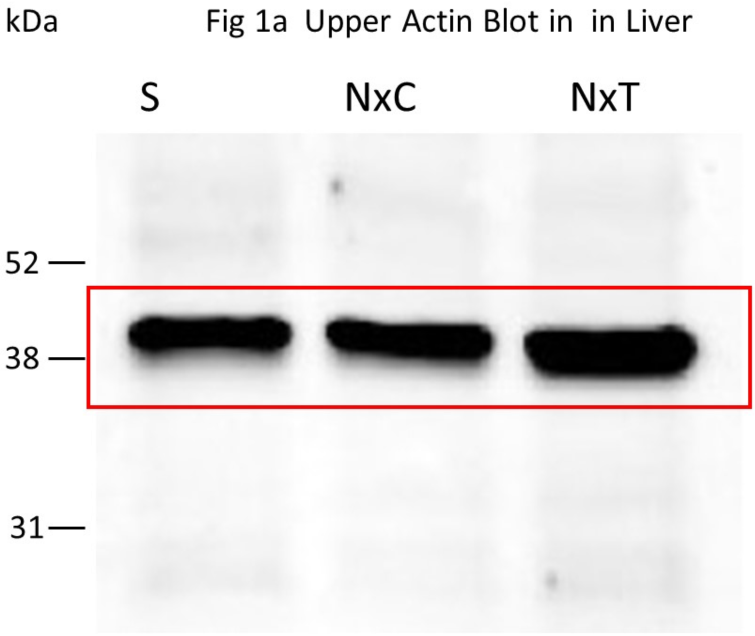

Fig 1d Plasma Albumin band stained with Ponceau Red

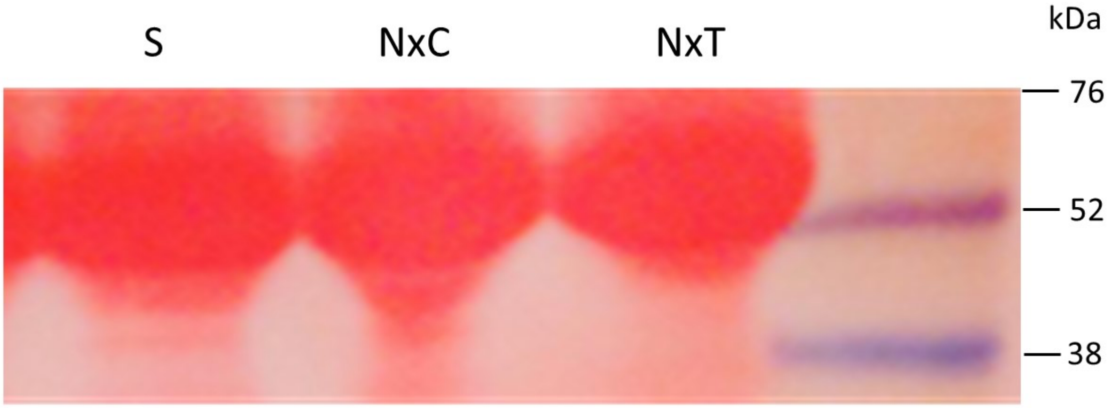

Fig 1d Plasma CSAD Blot

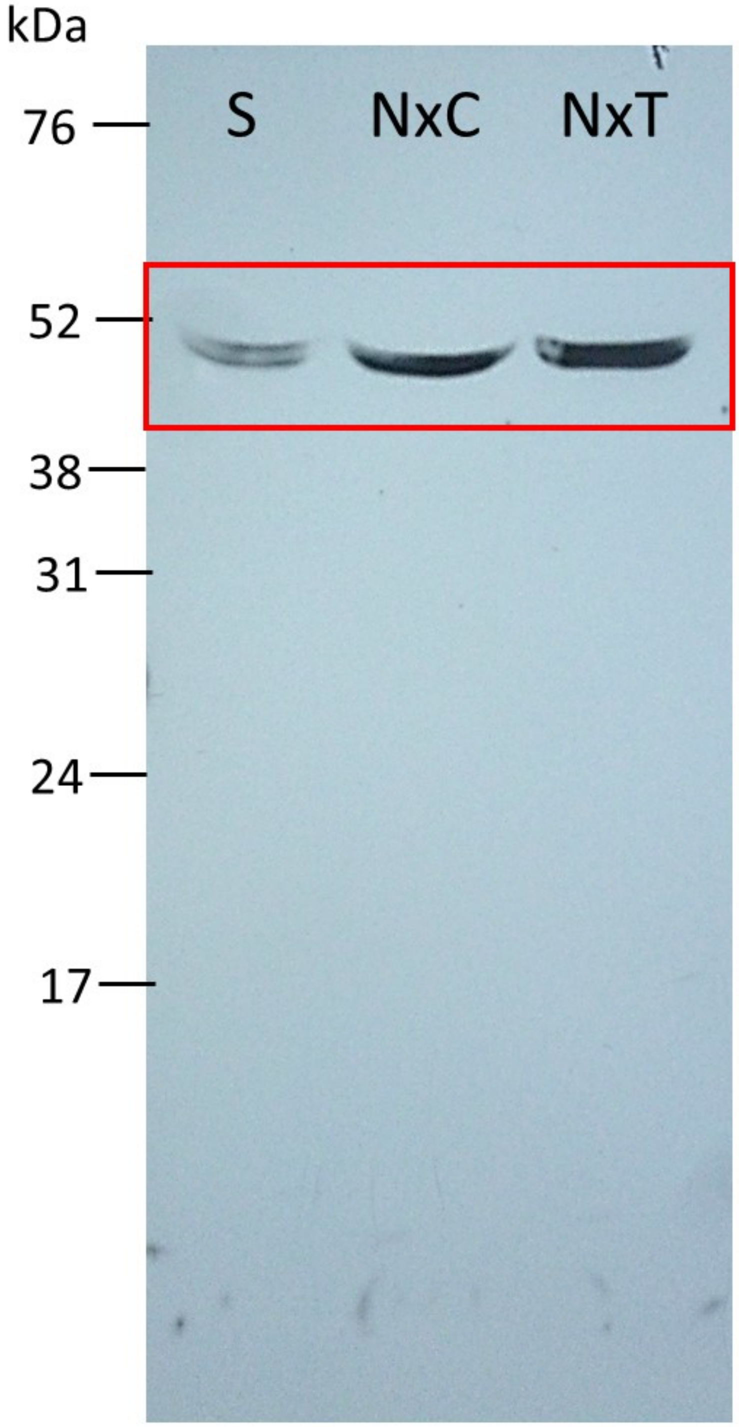

Fig 1e Liver CSAD & Blocking Peptide

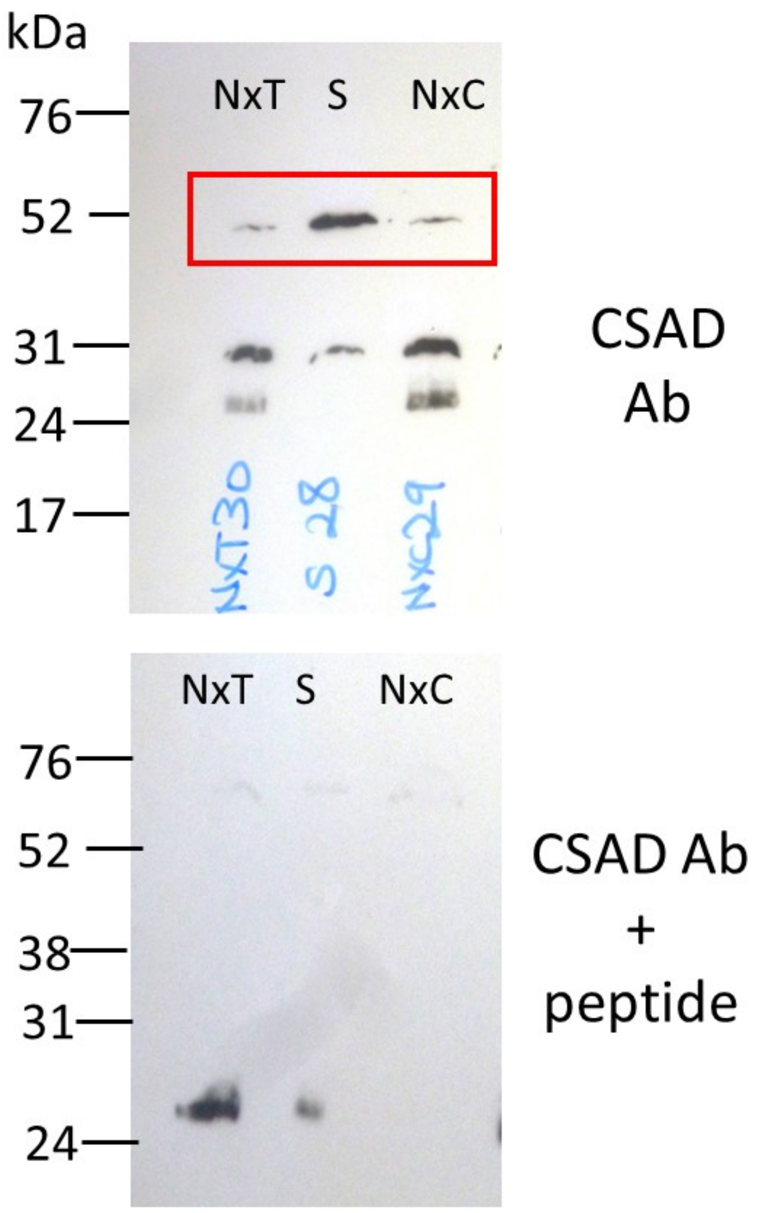

in liver lysates, the anti-CSAD antibody also detects a truncated ~ 30kDa CSAD fragment. This staining is specific (i.e. it is absent in the presence of blocking peptide) and it occurs in liver but not in plasma, possibly indicating that it is a degradation fragment generated within the hepatocytes.
